# Do Love You Me? Failure to Notice Word Transpositions is Induced by Parallel Word Processing

**DOI:** 10.5334/joc.335

**Published:** 2024-01-30

**Authors:** Joshua Snell, Alline Nogueira Melo

**Affiliations:** 1 Vrije Universiteit Amsterdam, Department of Experimental and Applied Psychology, Netherlands

**Keywords:** Reading, Word position coding, Transposed word effect, Syntax

## Abstract

Recent research has shown that readers may to fail notice word transpositions during reading (e.g., the transposition of “fail” and “to” in this sentence). Although this transposed word (TW) phenomenon was initially taken as evidence that readers process multiple words in parallel, several studies now show that TW-effects may also occur when words are presented one-by-one. Critically however, in the majority of studies TW-effects are weaker in serial presentation. Here we argue that while word position coding may to some extent proceed post-lexically (allowing TW-effects to occur despite seeing words one-by-one), stronger TW-effects in parallel presentation nonetheless evidence a degree of parallel word processing. We additionally report an experiment wherein a sample of Dutch participants (N = 34) made grammaticality judgments about 4-word TW sentences (e.g., ‘*the was man here*’, ‘*the went dog away*’) and ungrammatical control sentences (‘*the man dog here*’, ‘*the was went away*’), whereby the four words were presented either serially or in parallel. Ungrammaticality was decidedly more difficult to notice in the TW condition, but only when words were presented in parallel. No effects were observed in the serial presentation whatsoever. The present results bolster the notion that word order is encoded with a degree of flexibility, and further provide straightforward evidence for parallel word processing during reading.

## 1. Introduction

How do readers perceive word order? According to the OB1-reader model (Snell, van Leipsig, Grainger, & Meeter, 2018; Snell & Grainger, 2019a; Snell, Meeter, & Grainger, 2017), viewing a sequence of words prompts the activation of a spatiotopic representation that conveys the number of to-be-recognized words and their approximate lengths. Multiple lexical candidates are activated by the visual input (e.g., “*He was here*” activates ‘*he*’, ‘*was*’ and ‘*here*’ in parallel), and these are mapped onto locations in the spatiotopic representation, guided by visual cues (word length) and syntactic expectations.

As such, OB1-reader generates the prediction that readers may fail to notice word transpositions, such as the mistake in ‘*Do love you me?*’. Due to syntactic constraints, the parallel activation of ‘*love*’, ‘*you*’ and ‘*me*’ would lead ‘*you*’ to be mapped onto location 2 and ‘*love*’ onto location 3, resulting in the correct sentence ‘*Do you love me?*’.

The Transposed Word (TW) phenomenon, predicted by OB1-reader, was indeed observed experimentally, with a higher likelihood of failing to notice ungrammaticality when reading sentences that could be corrected (i.e., TW sentences such as ‘*the was man here*’ or ‘*the went dog away*’) than when reading sentences that could not be corrected (i.e., control sentences such as ‘*the dog man here*’ or ‘*the was went away*’) (Mirault, Snell, & Grainger, 2018; Snell & Grainger, 2019b; Liu, Li, & Wang, 2021; Snell, Mirault, Theeuwes, & Grainger, 2023). Besides providing a good test of the OB1-reader model, this TW phenomenon was more generally taken as evidence for parallel word processing, with the rationale that if words were strictly read and recognized one-by-one, readers would not fail to notice the error (e.g., after having recognized ‘*the dog…*’, the continuation ‘…*man*’ should signal an error).

However, against this initial rationale several research groups have recently produced a good counter-argument. Strikingly, it has been shown that TW-effects can also occur when sequences are presented word-for-word, thus precluding the parallel processing of multiple words (Liu, Li, Cutter, Paterson, & Wang, 2022; Huang & Staub, 2023; Milledge, Bhatia, Mensah-Mclead, *et al*., 2023; Hossain & White, 2023; see also Mirault, Vandendaele, Pegado, & Grainger, 2022, for a similar result). The alternative account of TW-effects provided by these authors is that word positions are flexibly encoded in a post-lexical buffer. As such, words may initially be processed serially (in the incorrect order), but by the time that the reader achieves conscious perception of word order, the ‘mistake’ has already been corrected.

The observation of TW-effects under serial presentation is theoretically interesting because it suggests that word position coding to a certain degree proceeds post-lexically. That is, a complete account of the word position coding process—thus far largely ignored by established models of reading—must involve some processing stage beyond the stage of word recognition.

In the present paper, however, we wish to argue that the overall pattern of results may nonetheless favor parallel word processing. The crucial point is that observations of TW-effects in either serial or parallel presentation alone do not inform the serial-versus-parallel processing debate. Rather, the critical test for an adjudication between theories is the *modulation* of TW-effects by presentation mode. In the remainder of this Introduction we outline why stronger TW-effects in parallel than serial presentation speak in favor of parallel word processing, and we take stock of the evidence obtained thus far. First, however, let us look at the serial processing account of these differences, as proposed by Huang and Staub (2023).

### 1.1 What can be inferred from interactions

Huang and Staub (2023) have proposed that, in parallel presentation, covert attention may shift to word N+1 during the fixation on word N. This would then slow down the integration (i.e., position coding) of word N. Meanwhile, processing of word N+1 starts earlier (i.e., prior to being fixated), which would speed up integration of word N+1. As a result, they argue, integration processes for word N and N+1 are more likely to overlap when words are presented in parallel, hence paving the way for more errors in word position coding even if serial processing were true.

We reckon the account of Huang and Staub (2023) is problematic for three reasons. Firstly, whichever processes delay or expedite word recognition should affect words N and N+1 equally. For instance, if covert shifting of attention to upcoming words typifies reading, then word N benefits from the same early processing as word N+1. The amount of processing time that is allotted to each word remains the same, irrespective of the distance between covert attention and the eye’s fixation; and so there is no reason to expect a higher degree of overlap in position coding when words are presented in parallel (under the assumption of serial processing).

Secondly, there is no reason why an attentional shift to word N+1 would slow down the integration of word N in the first place. The account of Huang and Staub is one where contextual integration happens for multiple words at once, while word identification is strictly serial. Therefore, claiming that the reading brain is a serial processor necessitates assuming that word identification and contextual integration are wholly independent processes. Any word that is being integrated is already fully recognized, so it really should not matter which word is being processed in the meantime.

Finally, the proposal by Huang and Staub would make the serial approach far less parsimonious. One would arrive at a scenario where parallel processing occurs at *all* levels of representation except the lexical level. Indeed, there is consensus on the fact that letter identities are processed across multiple words in parallel (e.g., Dare & Shillcock, 2013; Schotter & Payne, 2019; Snell & Grainger, 2019c; Snell, Vitu, & Grainger, 2017). If, additionally, the brain can handle higher-order contextual integration for multiple words in parallel, then the seemingly simpler intermediate process of parallel word identification would seem a given. At any rate, the serial account that is proposed by Huang and Staub is hardly serial.

Why, then, would stronger TW-effects in parallel than serial presentation suggest parallel word processing? If words were strictly processed one-by-one, then TW-effects must entirely hinge on the aforementioned post-lexical buffer. But the post-lexical buffer alone provides no explanation of why effects are larger when words are presented in parallel (barring accounts that are problematic for various reasons, as discussed above). If anything, TW-effects would be *diminished* in parallel presentation, given that readers have a chance to revisit words, thus allowing them more opportunities to spot the mistake. But if, in addition to employing the post-lexical buffer, the brain were to process multiple words in parallel, then the brain has to localize not just a single source of information, but multiple sources of information simultaneously. We deem it conceivable that the latter is cognitively more demanding. In short, we would argue that it is precisely because there is more information—and because the brain cannot wholly prevent itself from processing that information—that confusability is increased and TW-effects are enlarged when words are presented in parallel.

Hence, as we see it, the adjudication between theories may proceed relatively straightforwardly: a strictly serial processing model predicts equal TW-effects between serial and parallel presentation (because effects in both settings are solely driven by the post-lexical buffer) whereas a parallel processing model predicts stronger TW-effects in parallel presentation than in serial presentation (as the former setting induces consequences of parallel processing *in addition to* the post-lexical buffer).

By the above rationale, certainty about the modulation of TW-effects by presentation mode is a crucial next step. The good news is that these modulations are currently being explored, quantified and documented across labs and languages. The bad news is that the evidence obtained thus far isn’t entirely univocal. The majority of studies do show stronger TW-effects in the parallel presentation mode, in line with a parallel processing account (Liu et al., 2022; Mirault et al., 2022; Huang & Staub, 2023). Milledge et al. (2023) also observed stronger TW-effects in a parallel presentation mode, but only when compared against a serial presentation mode where each word appeared at the same central location (in line with all other studies under discussion here). Yet, in an alternative serial presentation setup where words’ spatial locations aligned with those of the parallel presentation, TW-effects were more or less equal to those obtained with parallel presentation.^1^ The study of Hossain and White (2023), meanwhile, provided no evidence for parallel word processing at all, as TW-effects were equal between the serial and parallel presentation mode—possibly because word exposure times were adjusted to individual reading speed in that particular study. In short, while the current state of affairs slightly favors a parallel processing account, the issue begs more evidence for better confidence.

### 1.2 The present study

Building on the recent studies of Liu et al. (2022), Mirault et al. (2022), Huang and Staub (2023), Milledge et al. (2023) and Hossain and White (2023), here we investigate TW-effects using a grammaticality judgment task with a serial versus parallel presentation of words, and, for the first time, using a sample of Dutch participants (N = 34). To preview results: strong TW-effects were observed in the parallel presentation condition, both in response times and accuracy. In contrast, no TW-effects were observed whatsoever in the serial presentation condition (in fact, numerical differences went in the opposite direction), and Bayesian statistics provided strong evidence for the null-hypothesis. All data and analysis scripts are available at https://osf.io/rs4kz/.

## 2. Methods

Thirty-four students from the Vrije Universiteit Amsterdam gave informed consent to their voluntary participation in this study for monetary compensation or course credit. All participants reported to be native to the Dutch language and non-dyslexic.

The experiment was a 3 × 2 factorial design with Grammaticality (Grammatical, Ungrammatical TW, Ungrammatical control) and Presentation (Serial, Parallel) as factors. As stimulus material we constructed 80 Dutch four-word sentences (with an average word length of ~3.76 letters) that, with a few manipulations, were used in all experimental conditions. Each sentence was presented twice in the Grammatical condition, once in the Ungrammatical TW condition and once in the Ungrammatical Control condition. The double presentation in the Grammatical condition was to have an equal number of Grammatical versus Ungrammatical trials. The Grammatical trials were merely used to induce the task, and were not included in our key analysis of the interaction between type of ungrammaticality (TW vs. Control) and presentation mode (Serial vs. Parallel). However, we do include Grammatical trials in analyses of main effects of presentation mode.

A TW version of each sentence was created by switching two adjacent words.^2^ Ungrammatical Control sentences were created by pairing grammatical sentences and switching two words within each pair (see, e.g., the examples in the Introduction). Using a Latin-square design with two groups we ensured that all sentences were shown in all conditions, but every individual participant saw each sentence only four times (one serial and one parallel presentation in the Grammatical condition, and one serial and one parallel presentation in either the Ungrammatical TW condition or the Ungrammatical Control condition). Words were presented in monotype font along the horizontal meridian, with each character subtending 0.3 degrees of visual angle. The total of 320 trials was presented in random order.^3^

**Figure 1:**
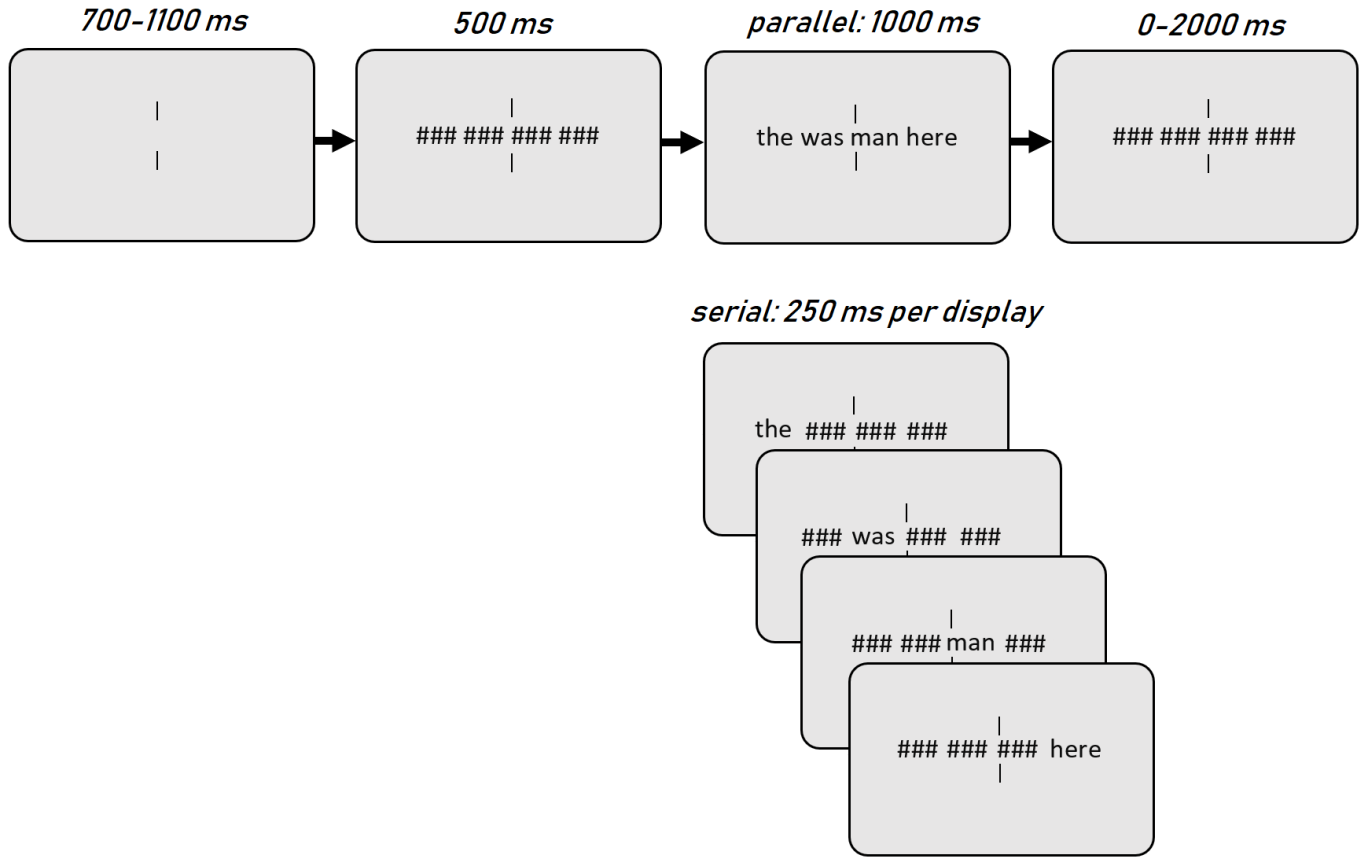
Schematic overview of the trial procedure. The size of stimuli relative to the screen is exaggerated in this example.

The experiment was implemented with OpenSesame (Mathôt, Schreij, & Theeuwes, 2012). A schematic overview of the trial procedure is shown in Figure 1. Each trial started with central vertical fixation bars for 700—1100 ms. Then a masked version of the stimulus (with four arrays of hashmarks instead of words) appeared for 500 ms. In the parallel presentation condition, the mask was replaced with the entire stimulus, which was shown for 1000 ms. In the serial presentation condition, the four masks were sequentially replaced by words, from left to right, with a 250 ms presentation time per word. As such we kept the two presentation modes as equal as possible both in terms of sentence exposure time as well as in terms of the availability of spatial information. Indeed, with the exception of Milledge et al.’s Experiment 3 (2023), all prior studies presented all words centrally in the serial presentation mode. We consider central RSVP to be problematic, because it omits all spatial cues to word order. Participants were instructed to respond as quickly and accurately as possible with the ‘/’ key for grammatical sentences and the ‘Z’ key for ungrammatical sentences. Responses were allowed as soon as the first word(s) appeared. Feedback was provided with a central green dot for a correct response, or a red dot in case of an incorrect response or having reached a time-out of 3000 ms. The experiment lasted approximately 20 minutes in total and participants were offered a break halfway through.

## 3. Results

As all participants had an accuracy over 75%, no participant was excluded from data analysis. Response times (RTs) and errors were analyzed with Linear Mixed-effect Models (LMMs) with Ungrammaticality (TW versus Control) and Presentation (Serial versus Parallel) as factors and items and participants as random effects (including both random slopes and random intercepts). Prior to the analyses of RTs, incorrectly answered trials (~12%) and trials that deviated more than 2.5 SD from the grand mean (a mere two trials) were removed. For all analyses we report *b*-values, standard errors (SEs) and *t*-values, whereby | *t* | > 1.96 was taken to indicate significance. Errors were analyzed with a generalized LMM, whereby | *z* | > 1.96 was taken to indicate significance. In case of an absence of effects, Bayesian statistical analyses were run to quantify evidence for the null-hypothesis.

**Figure 2:**
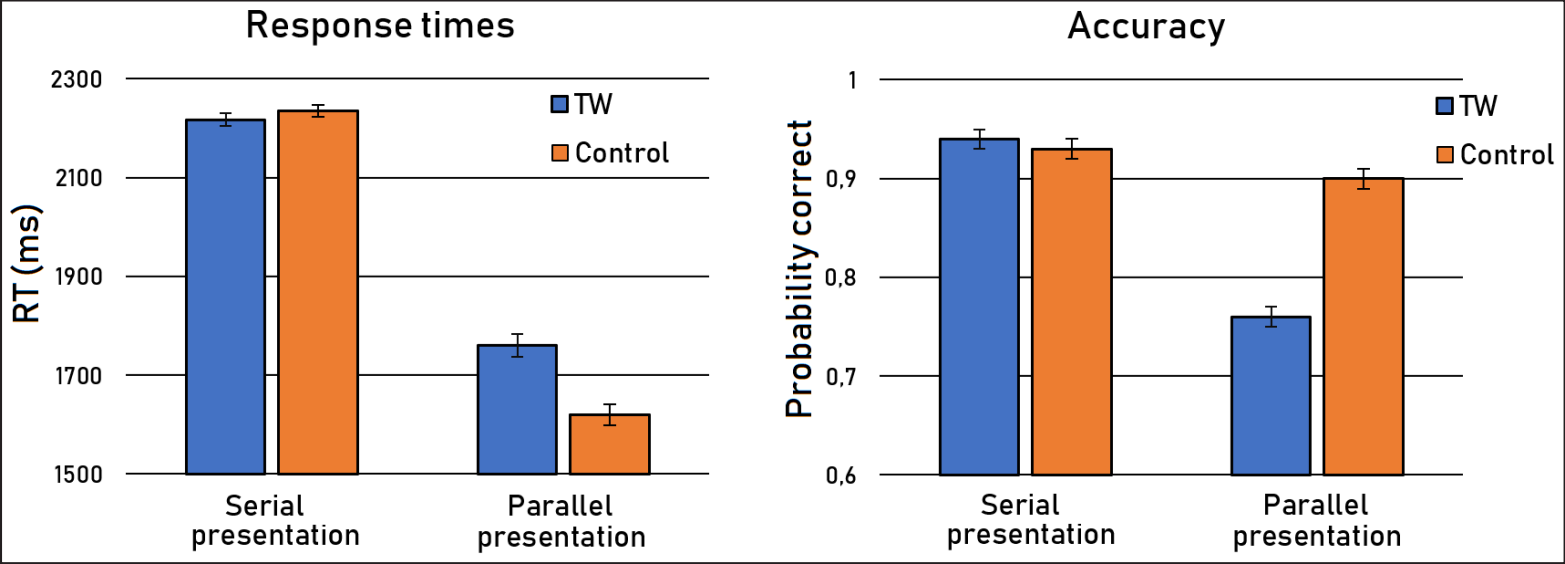
Average response times and accuracies. Error bars depict standard errors.

Results are presented in Figure 2.^4^ In line with previous research, ungrammaticality was significantly harder to detect in the case of TW sentences than control sentences. This was expressed both in RTs (*b* = 76.23, SE = 28.69, *t* = 2.66) and errors (*b* = 0.76, SE = 0.16, *z* = 4.65). We also observed a main effect of Presentation, with faster responses in Parallel than Serial presentation (*b* = 965.95, SE = 13.12, *t* = 73.64) but also a higher number of errors (*b* = 72, SE = 0.07, *z* = 10.39). Crucially, the TW-effect was modulated by Presentation, with a stronger effect in parallel than in serial presentation, again expressed both in RTs (*b* = 150.66, SE = 29.00, *t* = 5.20) and errors (*b* = 1.31, SE = 0.19, *z* = 7.01). In fact, the difference between serial and parallel presentation was so strong that the TW-effect was exclusively observed in parallel presentation (RTs: *b* = 119.30, SE = 39.03, *t* = 3.06; errors: *b* = 1.21, SE = 0.17, *z* = 7.32). No TW-effect was observed in serial presentation whatsoever (RTs: *b* = –15.31, SE = 32.20, *t* = –0.48; errors: *b* = –0.07, SE = 0.19, *z* = –0.39). These null-effects were accompanied by Bayes factors^5^ of 0.09 and 0.06 for RTs and errors, respectively, indicating strong evidence for the null hypothesis.

In a post-hoc analysis we assessed whether the critical point of ungrammaticality (PoU) influenced decisions. Snell and Grainger (2019b) have previously reported an *absence* of influences from the PoU, which was taken as additional evidence for parallel word processing at the time. Here we again found no main effects of PoU (RTs: *b* = 13.43, SE = 10.18, *t* = 1.32; errors: *b* = 0.02, SE = 0.05, *z* = 0.39), nor interactions between PoU and Presentation mode (RTs: *b* = 7.12, SE = 14.08, *t* = 0.51; errors: *b* = 0.05, SE = 0.09, *z* = 0.62), nor a moderating influence of PoU on TW-effects (RTs: *b* = 7.19, SE = 15.90, *t* = 0.45 ;errors: *b* = 0.09, SE = 0.08, *z* = 1.06).

## Discussion

We have argued that the failure to notice word transpositions during sentence reading suggests that readers process multiple words in parallel, whereby the order of simultaneously activated words is encoded with a degree of flexibility (e.g. Mirault et al., 2018; Snell et al., 2018; Snell & Grainger, 2019b). If the transposed word (TW) phenomenon were indeed solely driven by parallel word processing, then no TW-effects should be observed when words are presented in serial fashion. The present results straightforwardly suggest that this is the case. A clear TW-effect was observed for sequences presented in parallel, while a serial presentation of the same sequences produced no TW-effect whatsoever.

We must acknowledge, however, that absence of evidence is not evidence of absence (in spite of the use of Bayesian statistics). Importantly, the current results are at odds with other recent reports of TW-effects in serial presentation (e.g., Liu et al., 2022; Mirault et al., 2022; Huang & Staub, 2023; Milledge et al., 2023; Hossain & White, 2023). What might be causing this discrepancy? As argued in the Introduction, the observation of TW-effects in serial presentation per se suggests that word position coding may to a certain extent occur post-lexically. That we did not obtain evidence for the involvement of a post-lexical buffer this time around, then, may simply suggest that the contribution of this buffer in (flexible) word position coding is quite limited—and indeed limited to the extent that the buffer may at times not be used at all. With the exceptions of the study of Hossain and White (2023) and one of two experiments in the study of Milledge et al. (2023), all prior studies have shown stronger TW-effects in parallel presentation, indicating that word position coding *largely* unfolds online and during ongoing word identification. Adding the present results to the total body of evidence, the bigger picture thus far is more suggestive of TW-effects being driven by a degree of parallel word processing, than of TW-effects being solely driven by a post-lexical buffer.

Naturally, while we have not evidenced the involvement of a post-lexical buffer in word position coding here, that does not mean that this cognitive component bears no theoretical importance per se (see e.g., Gibson, Bergen, & Piantadosi, 2013; Levy, Bicknell, Slattery, & Rayner, 2009). It may well be, for instance, that TW-effects in serial presentation are more likely to occur when each word is viewed a shorter amount of time. Psychophysical studies may explore the precise contribution of the post-lexical buffer by systematically varying presentation times in a setup similar to that of the present study. We particularly take note of the study of Hossain and White (2023) here, as they adjusted word exposure times to individual reading speeds. This resulted in equal TW-effects between the serial and parallel presentation mode. We wager that the relative scarcity of information in their setup (due to shorter word exposure times) drove a larger reliance on the post-lexical buffer for word position coding. Nonetheless, the overall story remains that if multiple words are available simultaneously and without time limit—as is the case in natural reading—TW-effects are stronger, precisely because information is integrated across multiple words simultaneously.

In conclusion, here we have made a case for the theoretical significance of observations that transposed word effects are larger in parallel than in serial word presentation. The present results contribute to the growing body of evidence in this regard, and for the first time suggest that word position coding may also occur *exclusively* during ongoing word recognition, without involving a post-lexical buffer.

## Data Accessibility Statement

Data and analyses can be retrieved at https://osf.io/rs4kz/.

## Footnotes

One possible explanation for this is that the absence of spatial information with central RSVP induces a reliance on an ordinal—rather than spatial—representation of word order. The ordinal representation may be more robust against order confusion; hence the observation of strong TW-effects with distributed RSVP but not with central RSVP; (however, see Hossain & White, 2023).

Twenty sentences had a transposition of words 1 and 2; fourty-two sentences had a transposition of words 2 and 3; eighteen sentences had a transposition of words 3 and 4.

We had 2,720 measurements per condition for testing main effects, and 1,360 measurements per cell for testing interaction effects. Because stimuli were seen four times by each participant (in various conditions), and because trials were run in random order, we verified that the average number of previous occurrences of the same stimulus was equal across conditions. This was indeed the case: In serial presentation, TW: M = 1.58, SD = 1.28; Control: M = 1.56, SD = 1.33. In parallel presentation, TW: M = 1.51, SD = 1.28; Control: M = 1.61, SD = 1.30). Any difference in these numbers was statistically non-significant.

For the Grammatical filler trials, in Serial presentation, RT M = 2289 ms (SE = 5.67), Accuracy M = 0.95 (SE = 0.004); in Parallel presentation, RT M = 1327 ms (SE = 14.28), Accuracy M = 0.94 (SE = 0.004).

BF10 was computed with grammaticality as fixed factor and subjects and items as random effects, in the JASP statistical computing environment (JASP Team, 2023).
